# Exercise-induced irregular right heart flow dynamics in adolescents and young adults born preterm

**DOI:** 10.1186/s12968-021-00816-2

**Published:** 2021-10-21

**Authors:** Jacob A. Macdonald, Grant S. Roberts, Philip A. Corrado, Arij G. Beshish, Kristin Haraldsdottir, Gregory P. Barton, Kara N. Goss, Marlowe W. Eldridge, Christopher J. Francois, Oliver Wieben

**Affiliations:** 1 Radiology, Duke University, Durham, USA; 2 Medical Physics, University of Wisconsin-Madison, Madison, USA; 3 Pediatrics, University of Wisconsin-Madison, Madison, USA; 4 Medicine, University of Wisconsin-Madison, Madison, USA; 5 Biomedical Engineering, University of Wisconsin-Madison, Madison, USA; 6 Radiology, University of Wisconsin-Madison, Madison, USA

**Keywords:** Preterm, Premature, Cardiovascular magnetic resonance, 4D flow, Ventricle, Kinetic energy, Exercise

## Abstract

**Background:**

Preterm birth has been linked to an elevated risk of heart failure and cardiopulmonary disease later in life. With improved neonatal care and survival, most infants born preterm are now reaching adulthood. In this study, we used 4D flow cardiovascular magnetic resonance (CMR) coupled with an exercise challenge to assess the impact of preterm birth on right heart flow dynamics in otherwise healthy adolescents and young adults who were born preterm.

**Methods:**

Eleven young adults and 17 adolescents born preterm (< 32 weeks of gestation and < 1500 g birth weight) were compared to 11 young adult and 18 adolescent age-matched controls born at term. Stroke volume, cardiac output, and flow in the main pulmonary artery were quantified with 4D flow CMR. Kinetic energy and vorticity were measured in the right ventricle. All parameters were measured at rest and during exercise at a power corresponding to 70% VO_2max_ for each subject. Multivariate linear regression was used to perform age-adjusted term-preterm comparisons.

**Results:**

With exercise, stroke volume increased 10 ± 21% in term controls and decreased 4 ± 18% in preterm born subjects (p = 0.007). This resulted in significantly reduced capacity to increase cardiac output in response to exercise stress for the preterm group (58 ± 26% increase in controls, 36 ± 27% increase in preterm, p = 0.004). Elevated kinetic energy (KE_term_ = 71 ± 22 nJ, KE_preterm_ = 87 ± 38 nJ, p = 0.03) and vorticity (ω_term_ = 79 ± 16 s^−1^, ω_preterm_ = 94 ± 32 s^−1^, p = 0.01) during diastole in the right ventricle (RV) suggested altered RV flow dynamics in the preterm subjects. Streamline visualizations showed altered structure to the diastolic filling vortices in those born preterm.

**Conclusions:**

For the participants examined here, preterm birth appeared to result in altered right-heart flow dynamics as early as adolescence, especially during diastole. Future studies should evaluate whether the altered dynamics identified here evolves into cardiopulmonary disease later in life.

*Trial registration* None

**Supplementary Information:**

The online version contains supplementary material available at 10.1186/s12968-021-00816-2.

## Background

Preterm (or premature) birth, defined as birth at less than 37 weeks gestation, is a serious health problem, affecting 12% of all births and contributing to over 85% of complications and mortality in neonates [1]. To date, there is evidence that these individuals have increased risk for pulmonary [2], pulmonary vascular [3, 4], and systemic cardiovascular disease [5, 6].

Two recent studies have shown evidence of early right ventricular (RV)-pulmonary vascular uncoupling, a decreased ability of the RV to increase contractility or maintain stroke volume to preserve flow in the face of increased pulmonary arterial afterload, due to underlying preclinical pulmonary hypertension in adults born extremely preterm [4, 7]. This may be due in part to altered pulmonary microvascular structure and function as a result of underdeveloped cardiopulmonary systems at birth in the preterm neonate [8]. A study in more moderately preterm adults demonstrated impaired RV and pulmonary vascular function but coupling remained preserved [9]. Cardiovascular magnetic resonance (CMR) studies demonstrate that healthy adults born preterm have smaller RV volumes when compared to term controls [10, 11], which may further contribute to exercise limitations in this population.

There is a need for robust biomarkers to better understand the underlying mechanisms of cardiovascular disease risk in young adults born preterm, which may aid in early diagnosis and allow for monitoring of disease progression over time in an otherwise outwardly healthy population. 4D flow CMR holds promise for such a task, as it allows for the simultaneous characterization of blood flow dynamics in the main pulmonary artery (MPA) and RV. Notably, prior 4D flow studies have demonstrated that cardiac-remodeling associated with disease can be captured by parameters such as vorticity [12], which quantifies the rotation of blood in the ventricle, and kinetic energy (KE) [13], which describes the energy imparted to the blood by ventricular contraction and can be used to quantify the efficiency at which this energy is converted to the ejected stroke volume [14]. The utility of 4D flow has been further expanded by the recent development of free-breathing 4D flow CMR exercise challenges [15], which confers potential value in examining preterm populations, as this group has been shown to have increased exercise intolerance compared to term controls [16, 17]

In this study, a cohort of adolescents and young adults born preterm received cardiac 4D flow CMR examinations at rest and during submaximal supine exercise to better characterize underlying differences in right heart dynamics when compared to age-matched controls. We hypothesized that stroke volume augmentation in response to exercise would be impaired in the preterm population, in agreement with previous studies [10, 11], and the advanced 4D flow analysis would reveal previously unreported dysfunction induced by abnormal ventricular flow dynamics in preterm subjects, thereby improving our understanding of this unique phenotype.

## Methods

### Subject population

This study was approved by the local Institutional Review Board and is compliant with the Health Insurance Portability and Accountability Act. Eleven preterm young adults (27 ± 1 years; 5 male,) and 17 preterm adolescents (13 ± 1 years; 6 male) were recruited. These subjects were drawn from the Newborn Lung Project (NLP) cohort [18] at the University of Wisconsin-Madison. The NLP cohort included individuals born prior to 32 weeks of gestation at a birthweight below 1500 g. Invitations to participate in this study were emailed to cohort members or their guardians with up-to-date contact information and residence in Wisconsin. Approximately 10% of those contacted responded and participated in phone interviews which provided further details on the study and determined if the individual met any specific exclusion criteria. Exclusion criteria included any history of cardiovascular or cardiopulmonary disease, exclusive of neonatal comorbidities such as bronchopulmonary dysplasia or resolved pulmonary hypertension, any physical limitations inhibiting exercise capabilities, and any other significant health problems that required daily medication use. Qualified individuals were given the chance to accept or decline participation in this study at this point. Eleven young adults (26 ± 1 years; 8 male) and eighteen adolescents (13 ± 1 years; 8 male) born at term were also recruited to serve as age-matched healthy controls. All subjects were screened with a Global Physical Activity Questionnaire (GPAQ) [19] or a Physical Activity Questionnaire for older Children (PAQ-C) [20] to ensure there were similar levels of physical activity between the preterm groups and the age-matched control groups.

### Imaging and reconstruction

All imaging was performed on a clinical 3T CMR scanner (Discovery 750, General Electric Healthcare, Waukesha, Wisconsin, USA) with an 8-channel cardiac coil between March 2016 and November 2017. 4D flow acquisitions were performed at rest and during exercise with a radially-undersampled, 5-point encoded PC VIPR [21, 22] sequence with the following parameters: TR/TE = 6.2/2.0 ms, flip angle = 10°, velocity encoding (venc) = 200 cm/s, field of view = 32 × 32 × 32 cm, acquired spatial resolution = 1.25 mm isotropic, scan duration = 9.25 min. The imaging volume covered the entire upper torso, including the heart and great vessels. Acquisitions were free-breathing with retrospective electrocardiogram (ECG) gating and respiratory gating from an abdominal belt. Average heart rate during imaging was calculated from the ECG gating files recorded on the scanner.

As a result of increased motion during exercise and reduced reliability of ECG leads with perspiration, there was an increase in missed ECG triggers for many subjects during exercise. Unreliable gating profiles resulted in inaccurate retrospective temporal binning of projections, leading to reconstructed flow waveforms with erroneous increases in diastolic volumetric blood flow rates. To counter this, a novel ECG gating correction algorithm was applied to compromised gating files [23]. Correction was performed if more than 5% of the acquired projections were acquired at a cardiac time longer than the median RR interval length (i.e., outside of the expected cardiac cycle length). The details of this algorithm are presented in an additional file (see Additional file 1).

Images were reconstructed offline and only used data acquired during expiration identified through adaptive thresholding of the respiratory waveform at 50% efficiency [24]. The number of reconstructed cardiac phases was kept to fifteen to compensate for minor signal-to-noise (SNR) losses expected with exercise [15] with an increased number of projections per phase.

### Exercise protocol

**Fig. 1:**
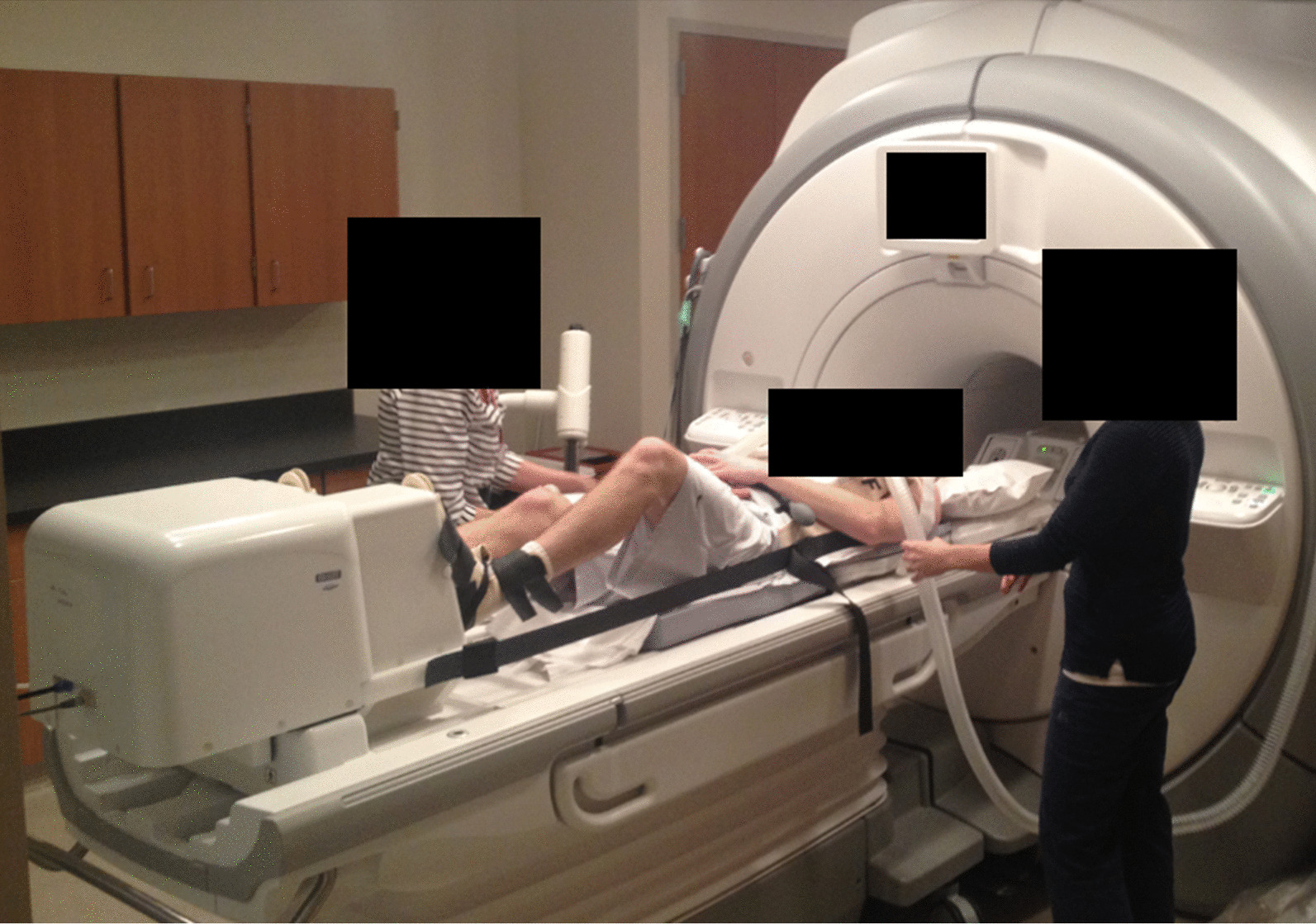
Sample setup for exercise imaging. The subject lies in a supine position and steps against the pneumatic pedals of the cardiovascular magnetic resonance (CMR)-compatible exercise stepper (seen at the end of the CMR bed). The pressure supplied to the pedals control resistance. The subject is connected to the stepper with Velcro straps around the feet and a harness around the chest to minimize bulk motion during exercise

The exercise power corresponding to each subject’s VO_2max_ (maximal oxygen uptake) was established on an upright bicycle ergometer in an exercise laboratory as previously described [16]. For exercise imaging, each subject exercised in a supine position in the scanner bore with a commercial CMR-compatible recumbent stepper (Cardio Stepper Module, Ergospect GmbH, Innsbruck, Austria). An example of the setup is shown in Fig. 1. Subjects were verbally coached to step at a cadence of 60 steps/min at 70% of the power corresponding to their VO_2max_ for the duration of imaging. Exercise imaging began after three minutes of stepping to allow subjects to achieve a steady-state heart rate. The stepper automatically adjusted the stepping resistance in response to changes in stepping cadence to maintain the target power setting.

### Image analysis

Background phase correction for the velocity maps was performed on all datasets by fitting a 3^rd^ order polynomial to phase in stationary tissue, defined by semi-automatically thresholding time-averaged magnitude and velocity data [25]. To assess right heart flow, time-averaged complex difference 4D flow datasets were processed using MIMICS (version 17.0, Materialize, Leuven, Belgium) to segment the heart and great vessels. Two-dimensional measurement planes were placed orthogonal to the main pulmonary artery in Ensight (Version 10.0, Ansys, Canonsburg, Pennsylvania, USA). A custom MATLAB (The Mathworks Inc., Natick, Massachusetts, USA) tool [26] was used to identify the vessel walls and integrate velocity across the vessel lumen to quantify blood flow over the cardiac cycle. Stroke volume was calculated by integrating flow through the MPA over the reconstructed cardiac cycle. Cardiac output was calculated by multiplying stroke volume (SV) by heart rate (HR). SV and cardiac output were both indexed by subject body surface area.

To quantify ventricular KE, the RV was segmented from time-averaged magnitude 4D flow images with MIMICS. A MATLAB script applied this segmentation mask to the time-resolved velocity images to extract the velocity magnitude for each voxel included in the RV mask. Ventricular KE was calculated using the following formula:

$$KE=\sum_{i}^{voxels}\frac{1}{2}m{v}_{i}^{2}=\frac{1}{2}\rho V\sum_{i}^{voxels}{v}_{i}^{2}$$

where *ρ* = 1060 kg/m^3^ is the assumed density of blood, *V* = 1.95 mm^3^ is the voxel volume, and *v*_*i*_ is the velocity magnitude for voxel *i*. RV energy “efficiency” was defined as the SV normalized by ventricular KE, *η* $=\frac{SV}{KE}$. To further characterize the behavior of the intra-ventricular velocity fields, vorticity was calculated for each voxel in the segmented RV by taking the curl of the velocity field. Total vorticity was determined by adding all voxels included in the mask. KE, *η*, and vorticity were calculated for all cardiac phases. Peak systolic and diastolic values for these parameters were defined as the local maximum values on the time-resolved curves during the cardiac phases associated with systole and diastole, respectively. These measurements were indexed by mask size.

To lend further context to the quantitative vorticity measurements, streamline and pathline visualizations were generated in Ensight to qualitatively assess differences in intra-ventricular flow. The streamlines and pathlines were generated from a plane bisecting the tricuspid valve, RV apex, and pulmonary valve to characterize the main flow patterns through the ventricle. The flow dynamics of subjects with abnormal vorticity measurements were inspected to identify differences in flow patterns compared to representative controls. Special attention was given to the structure of the diastolic filling vortex, which has been shown to be important to efficient RV function [27].

### Statistical analysis

All measurements are presented as the mean of the samples plus/minus standard deviation. To further enhance the statistical power and allow for age-adjusted comparisons, the two age groups were combined, and multivariate linear regression was performed with age and birth status as independent variables. Term/preterm status was represented as a dummy variable. Cardiac index, SV index, systolic KE, diastolic KE, systolic *η*, diastolic *η*, systolic vorticity, and diastolic vorticity were included in the model as dependent variables. Significant differences between groups was determined from the t-statistic of a hypothesis test with the null hypothesis that the birth status coefficient was equal to zero. A significance threshold of α = 0.05 was used for all tests. p-values were uncorrected for multiple comparisons. While this confers an increased risk for type I error, it reduces type II error, which we deemed was appropriate in this attempt to characterize subtle differences in flow dynamics between two overtly healthy cohorts.

## Results

Fifty-two out of 57 subjects successfully completed both rest and exercise imaging. Two term adults and one preterm adult were unable to complete both 4D flow exams due to claustrophobia or general discomfort. One term child and one preterm child stopped exercising during imaging due to trouble concentrating for the entire scanning session. Anthropomorphic metrics, baseline ventricular volumetry [11], and exercise measurements for the subjects that completed imaging in each group are given in Table 1. Of the preterm subjects who successfully completed both imaging sessions, 2 adolescents and 5 adults were born prior to 28 weeks of gestation. Five preterm adults and seven preterm adolescents had been diagnosed with neonatal bronchopulmonary dysplasia.

**Table 1:** Anthropomorphic metrics, baseline ventricular volumetry, and exercise measurements for subjects included in final analysis

	Term adults (n = 9)	Preterm adults (n = 10)	p_adult_	Term adolescents (n = 17)	Preterm adolescents (n = 16)	p_adolescent_
Anthropomorphic metrics						
Sex	6M, 3F	5M, 5F	–	8M, 9F	5M, 11F	–
Current age [year]	26 ± 1	27 ± 1	0.06	13 ± 1	13 ± 1	0.70
Gestational age [week]	40 ± 1	29 ± 3	**< 0.001**	40 ± 1	28 ± 2	**< 0.001**
Birth weight [g]	N/A*	1087 ± 297	–	3497 ± 366	1097 ± 274	**< 0.001**
Current height [m]	1.74 ± 0.07	1.69 ± 0.12	0.28	1.64 ± 0.09	1.58 ± 0.10	0.08
Current weight [kg]	69 ± 9	67 ± 15	0.71	51 ± 10	48 ± 9	0.56
BMI	22.7 ± 1.6	23.3 ± 3.3	0.64	18.6 ± 2.3	19.4 ± 2.7	0.42
BSA [m^2^]	1.83 ± 0.15	1.77 ± 0.24	0.53	1.51 ± 0.19	1.45 ± 0.17	0.37
RV volumetry						
EDVI [mL/m^2^]	97 ± 15	93 ± 10	0.48	81 ± 11	71 ± 12	**0.01**
ESVI [mL/m^2^]	43 ± 9	41 ± 8	0.42	36 ± 6	29 ± 6	**0.004**
SV index [mL/m^2^]	54 ± 9	52 ± 6	0.73	45 ± 7	42 ± 7	0.12
EF	0.55 ± 0.05	0.57 ± 0.05	0.59	0.56 ± 0.05	0.58 ± 0.04	0.09
Exercise measurements						
VO_2max_ [L/min] [4, 28]	3.5 ± 0.7	2.6 ± 0.6	**0.008**	2.5 ± 0.5	2.0 ± 0.5	**0.007**
VO_2max_ [mL/kg/min] [4, 28]	50.0 ± 10.4	38.1 ± 8.6	**0.01**	48.3 ± 11.0	43.3 ± 6.9	0.13
P_max_ [W]	231 ± 54	184 ± 42	**0.05**	153 ± 34	124 ± 27	**0.01**
GPAQ [MET/week] [4]	3368 ± 2550	3420 ± 2006	0.96	–	–	–
PAQ-C [29]	–	–	–	1.93 ± 0.39	1.89 ± 0.45	0.79

Results are presented as the mean plus/minus the standard deviation of the sample. p-values are for comparisons within each age cohort. Significant p-values (p < 0.05) are in bold. *Birth weights were not collected for the young adult term controls. *M* male, *F* female, *BMI* body mass index, *BSA* body surface area, *EDVI* end diastolic volume index, *ESVI* end systolic volume index, *SV* stroke volume, *EF* ejection fraction, *VO*_*2max*_ maximum oxygen consumption, *P*_*max*_ maximum exercise power, *GPAQ* Global Physical Activity Questionnaire, *PAQ-C* Physical Activity Questionnaire for older Children

The mean exercise power during imaging was 153 ± 36 W for term adults, 131 ± 24 W for preterm adults, 107 ± 24 W for term adolescents, and 87 ± 19 W for preterm adolescents. Both the term adults and adolescents exercised at significantly higher powers than the age-matched preterm subjects (adults: p = 0.04; adolescents: p = 0.005).

**Fig. 2:**
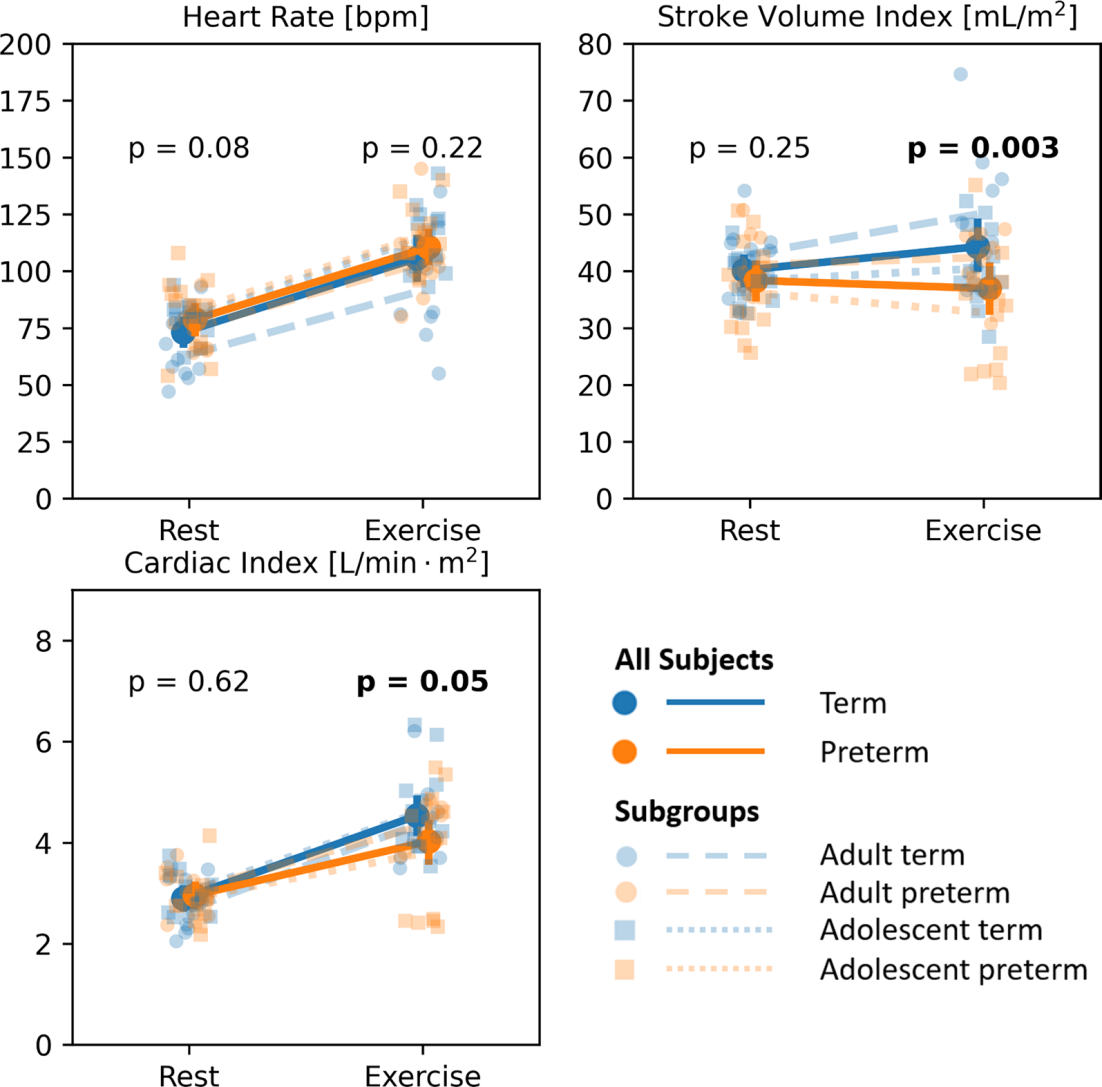
Changes in heart rate, stroke volume index, and cardiac index with exercised as measured with 4D flow in the main pulmonary artery for term (blue) and preterm (orange) subjects. p-values represent the significance of differences between term and preterm subjects at rest and during exercise

Measured hemodynamic parameters for term and preterm born groups at rest and exercise are presented in Table 2. The mean percent change in these parameters with exercise are included. Figure 2 shows the measured distributions for heart rate (HR), stroke volume (SV), and cardiac index (CI) as measured in the MPA at rest and during exercise.

**Table 2:** Hemodynamic parameters at rest and during exercise with corresponding mean percent change

Parameter	Rest			Exercise			Mean percent change [%]		
	term	Preterm	p	Term	Preterm	p	Term	Preterm	p
HR [bpm]	73 ± 13	79 ± 14	0.08	106 ± 22	110 ± 16	0.22	44 ± 14	42 ± 23	0.80
SV index [mL/m^2^]	40 ± 5	38 ± 7	0.25	44 ± 10	37 ± 10	**0.003**	10 ± 21	-4 ± 18	**0.007**
CI [L/min]	2.9 ± 0.5	3.0 ± 0.5	0.62	4.5 ± 0.8	4.0 ± 1.0	**0.05**	58 ± 26	36 ± 27	**0.004**
Systolic KE × 10^–8^ [J]	9.2 ± 3.6	10.3 ± 3.0	0.30	14.4 ± 5.1	12.9 ± 4.5	0.31	64 ± 61	30 ± 43	**0.04**
Diastolic KE × 10^–8^ [J]	7.1 ± 2.2	8.7 ± 3.8	**0.03**	12.5 ± 5.6	13.7 ± 6.6	0.45	80 ± 73	66 ± 61	0.44
Systolic η [mL/mJ]	11.5 ± 3.3	9.7 ± 4.4	0.31	8.6 ± 4.4	6.9 ± 2.1	0.15	− 20 ± 30	− 20 ± 32	0.99
Diastolic η [mL/mJ]	13.7 ± 2.5	11.7 ± 4.4	0.07	10.2 ± 4.4	6.8 ± 2.3	**0.002**	− 26 ± 27	− 37 ± 24	0.20
Systolic ω [s^−1^]	87 ± 26	98 ± 30	0.14	126 ± 37	113 ± 31	0.26	50 ± 46	18 ± 21	**0.006**
Diastolic ω [s^−1^]	79 ± 16	94 ± 32	**0.01**	102 ± 20	116 ± 34	0.08	32 ± 28	27 ± 21	0.34

Results are presented as the mean plus/minus the standard deviation of the sample. p-values are provided for comparisons between term and preterm subjects with an age-adjusted model. Significant p-values (p < 0.05) are in bold. *HR* heart rate, S*V* stroke volume, *CI* cardiac index, *KE* mean kinetic energy, *η* kinetic energy efficiency, *ω* mean vorticity

Preterm subjects showed significantly lower SV and CI compared to term subjects during exercise (p = 0.003 and p = 0.05 respectively). This was driven by what appeared to be an inability to increase SV (and CI by extension) with exercise. While increases in HR with exercise was similar between term and preterm groups (p = 0.80), preterm subjects averaged a decrease in SV of 4% with exercise in comparison to the modest average increase of 10% in term subjects (p = 0.007).

**Fig. 3:**
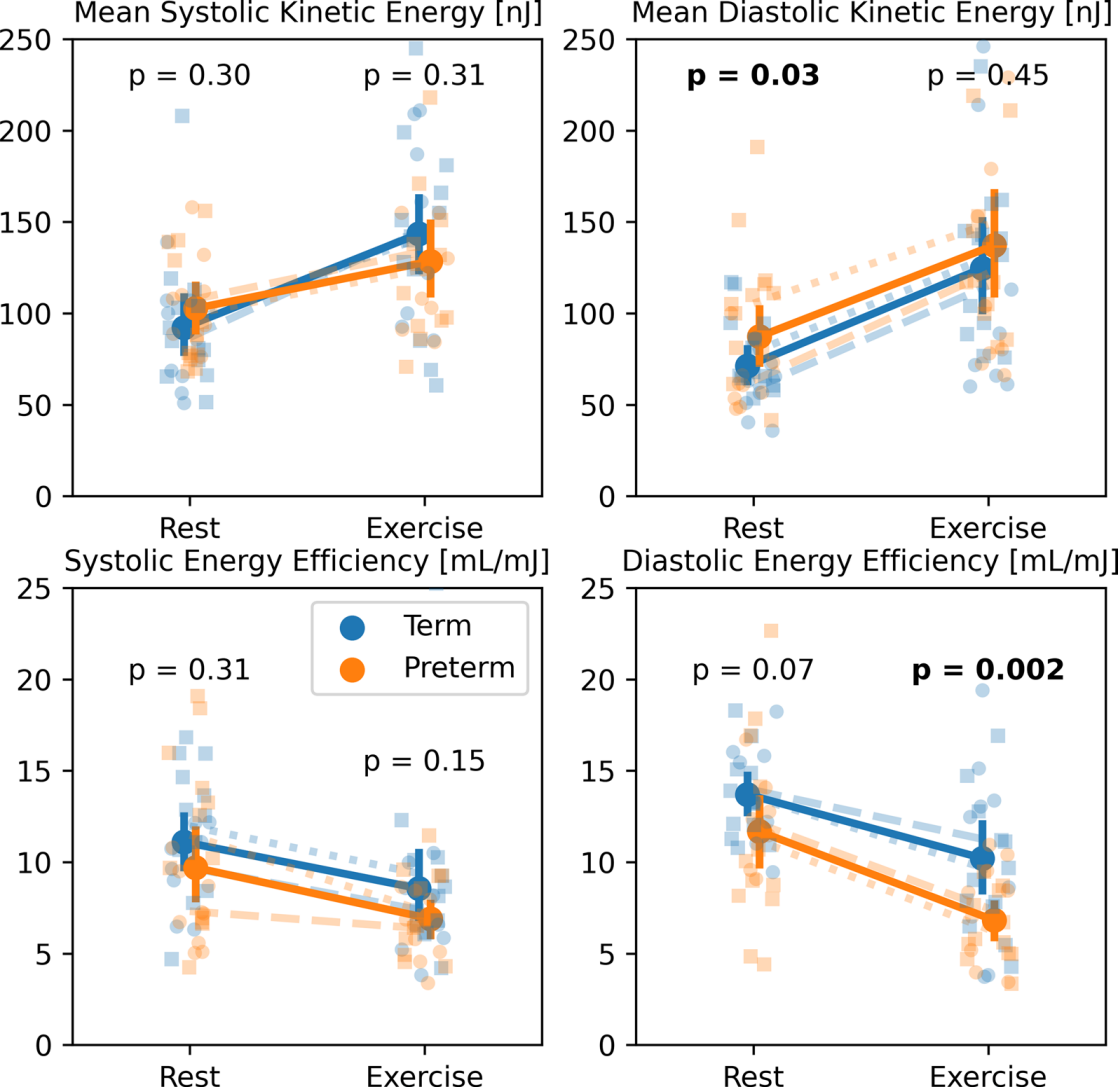
Changes in mean peak systolic kinetic energy, mean peak diastolic kinetic energy, systolic energy efficiency, and diastolic energy efficiency with exercise as measured with 4D flow in the right ventricle for term (blue) and preterm (orange) subjects. p-values represent the significance of differences between term and preterm subjects at rest and during exercise. Refer to Fig. 2 for a more detailed legend

Figure 3 shows the measured distributions for peak systolic KE, peak diastolic KE, systolic energy efficiency, and diastolic energy efficiency as measured in the RV for all subject groups at rest and during exercise. At rest, preterm subjects had significantly higher KE during diastole (p = 0.03). Diastolic energy efficiency was lower on average in the preterm subjects at both rest (p = 0.07) and during exercise (p = 0.002). Term subjects averaged significantly larger increases in systolic KE in response to exercise (p = 0.04) with minimal changes in this parameter in the preterm population.

**Fig. 4:**
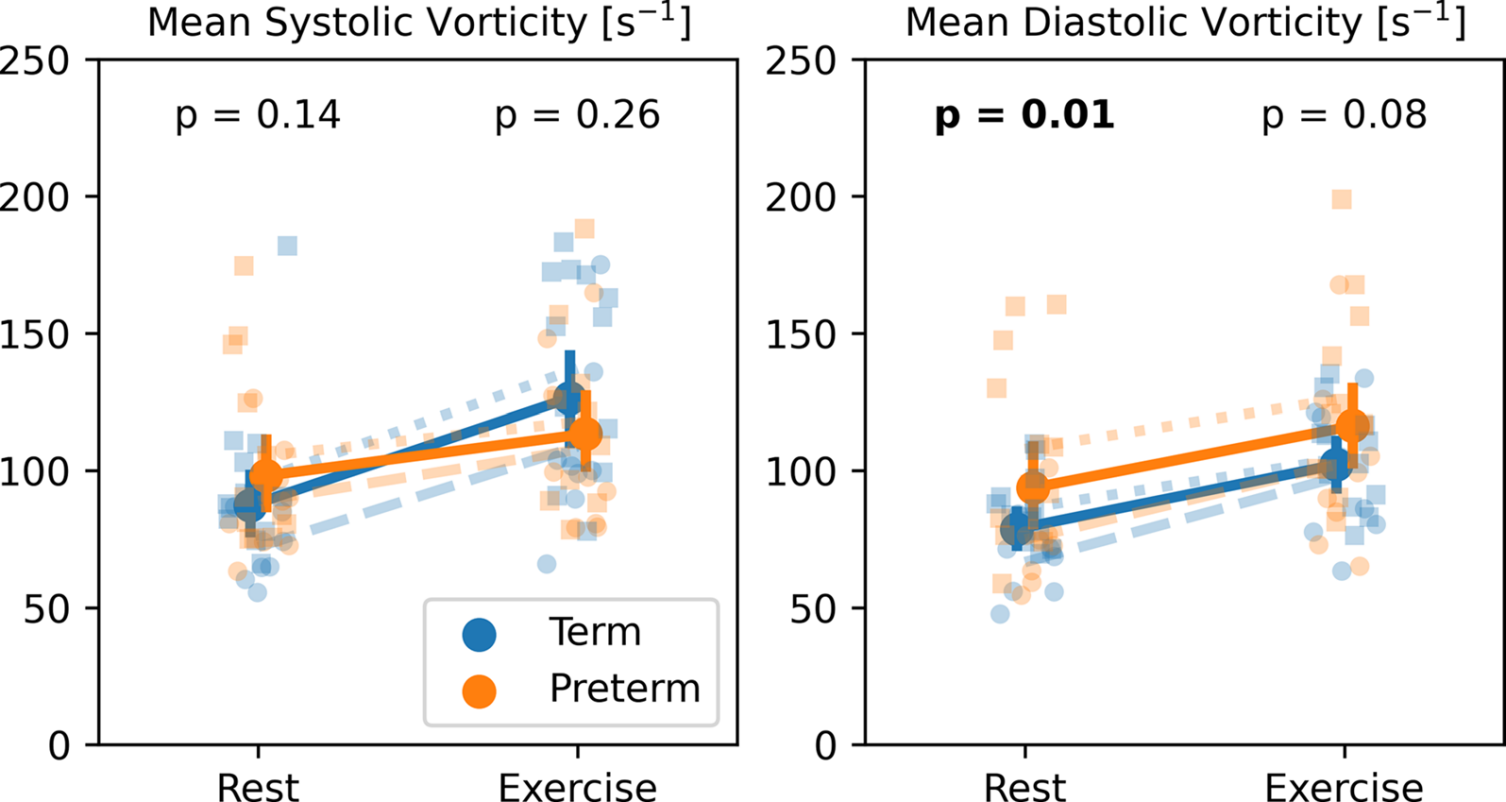
Changes in peak systolic vorticity and peak diastolic vorticity with exercise for term (blue) and preterm (orange) subjects. p-values represent the significance of differences between term and preterm subjects at rest and during exercise. Refer to Fig. 2 for a more detailed legend

Figure 4 shows the measured distributions for systolic vorticity and diastolic vorticity as measured in the RV for all subject groups at rest and during exercise. Mean diastolic vorticity measurements were elevated in preterm subjects compared to term controls significantly at rest (p = 0.01) and non-significantly during exercise (p = 0.08). Term controls showed significantly larger increases in systolic vorticity in response to exercise (p = 0.006).

**Fig. 5:**
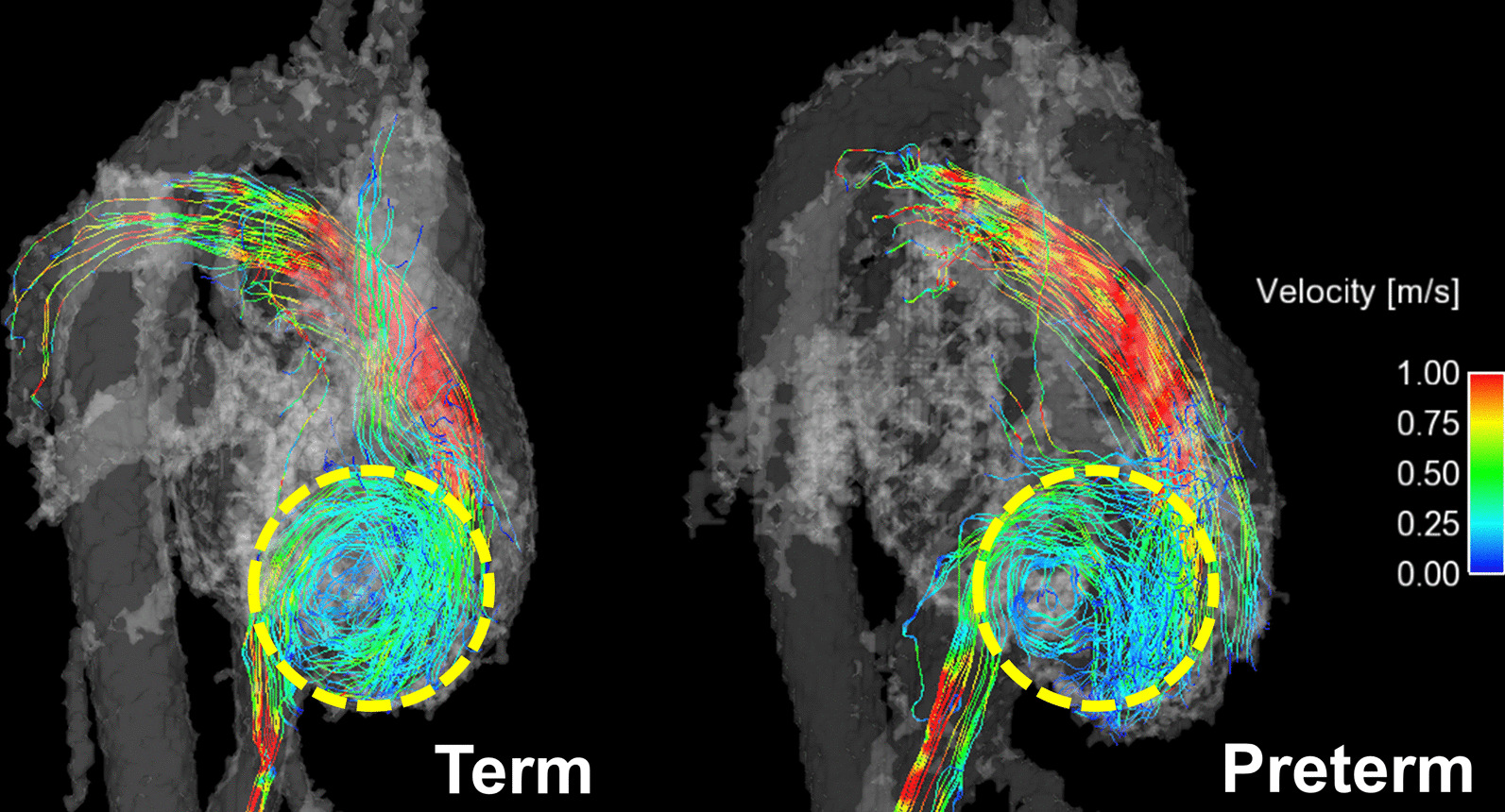
Representative right heart streamline visualizations of the entire cardiac cycle in term and preterm subjects during exercise. As indicated in the regions enclosed by the dashed yellow circles, term subjects have clearly structured, circular diastolic filling vortices. Diastolic filling vortices are more chaotic and incoherent in preterm subjects

The streamline visualizations for representative term and preterm subjects in Fig. 5 provide some explanation of the higher diastolic vorticity in the preterm born group. The diastolic filling vortices featured a prominent circular structure in term subjects. Filling vortices in preterm subjects were less structured and appeared to contain more chaotic flow, yielding a larger result in the curl operation of the vorticity calculation. Additional movies are included showing pathline animations in the RV of another set of representative term and preterm subjects (see Additional file 2 for term and Additional file 3 for preterm). The same phenomena as described in Fig. 5 can be observed in these videos.

## Discussion

Exercise testing remains an invaluable tool that provides diagnostic and prognostic information for at-risk patients and patients with overt cardiovascular and pulmonary disease [30, 31]. When combined with exercise, imaging modalities like CMR have the potential to offer greater diagnostic accuracy, provide additional information regarding cardiac structure and function, and improved prognostication. Given our previous findings of smaller biventricular chambers [11], perturbed pulmonary vascular function [4, 7, 8], and exercise intolerance [17], we hypothesized that individuals born very to extremely preterm would have an impaired RV response to exercise. We sought out to answer this question by utilizing in-bore submaximal exercise combined with 4D flow CMR to better understand whether altered ventricular hemodynamics played a role that might provide insight into the previous findings. The major findings of this study were preterm born adolescents and adults exhibit a blunted SV response to exercise which may have been related to altered diastolic hemodynamics (i.e., vorticity and kinetic energy).

As hypothesized, preterm subjects showed impaired stroke volume augmentation with exercise driven by highly significant differences between subject groups during exercise. The lack of a significant difference at rest was likely a function of reduced statistical power from a smaller sample size in this study and the fact that SV was calculated with phase contrast CMR in this study rather than the gold-standard cine balanced steady state free precession (bSSFP) approach. Notably, many preterm subjects showed decreased SV with exercise. While a blunted SV response to exercise has been associated with both adolescence and supine exercise [32, 33] (and was observed here in the relatively modest increases in SV term subjects showed with exercise), preterm subjects showed a further attenuated response. This reduced SV during exercise stress mirrored prior findings with stress echocardiography [34], thoracic bioimpedance [17], and invasive catheterization [4]. The reliance on increasing only HR to raise cardiac output in preterm subjects is concerning, as it is consistent with that observed in many right-heart diseases, such as pulmonary artery hypertension [35], and may suggest reduced cardiac reserve in these subjects.

As hypothesized, KE and vorticity measurements coupled with qualitative streamline visualizations revealed subtle differences in intraventricular flow in the preterm subjects. Minimal increases in systolic KE in preterm subjects compared to term controls suggested reduced capacity to properly respond to the demands of exercise stress, similar to the response seen in SV, which combined may suggest reduced ventricular-vascular coupling [7]. Exercise energy efficiency measurements were significantly lower during diastole in preterm born subjects. It is important to note, however, that this significance may be driven by the significant difference in SV observed between term and preterm subjects. The lower KE efficiency during diastole in the preterm subjects was consistent with myocardial strain measurements reported from bSSFP acquisitions in the same subjects. These measurements indicated hyper-contractility in preterm subjects (greater strain to produce the same SV) and a slower recovery to baseline strain during diastole [11].

The increased KE and vorticity observed in the preterm group was likely related to the observed inefficiency of the diastolic filling vortices. Pasipoularides et al. have previously hypothesized that diastolic filling vortices are an important mechanism for dissipating excess KE entering the ventricle [27]. If inflowing kinetic energy is not properly dissipating, it may contribute to an inflow-impeding convective pressure rise in the ventricle. Such a pressure increase could account for the hypercontractility and decreased energy efficiency observed in the preterm subjects.

Put together, these factors suggest a degree of abnormal intracardiac flow in adolescents and young adults born that may explain decreased exercise tolerance and reduced cardiac functional reserve. This warrants further investigation given the young age of these subjects and their lack of overt cardiopulmonary disease. If sustained, altered flow can lead to increased stresses on the myocardium and lead to ventricular remodeling, a precursor to cardiac disease. Longitudinal studies with larger sample sizes could play a valuable role in better understanding how the flow patterns reported here evolve with age in individuals with a history of preterm birth.

### Limitations

The approach used to quantify KE and vorticity in this study has some inherent limitations. RV volumes were segmented from time-averaged 4D flow magnitude images. This approach suffers from reduced myocardium-to-blood-pool contrast and lack of dynamic information when compared to cine bSSFP-based segmentation [36]. KE measurements using time-averaged 4D flow magnitude segmentations have been shown to suffer from reduced repeatability compared to flow measurements in the MPA [15]. While resting bSSFP images could be registered to resting 4D flow data to improve ventricle segmentation, this approach was not feasible during exercise due to the incompatibility of the breath-hold requirement for bSSFP imaging during exercise. In addition to segmentation challenges, the high VENC employed in this study was selected to characterize high velocities in the MPA and was not optimized for slower ventricular flow. This likely contributed to increased noise in KE and vorticity measurements. Dual-VENC approaches [37] may allow for optimized velocity encoding in the great vessels and cardiac chambers but would almost double scan times. Finally, while the decision to reconstruct 15 cardiac phases helped offset SNR loss during exercise, it increased the apparent temporal resolution, reducing sensitivity to the early diastolic motion important for properly characterizing diastolic dysfunction.

## Conclusion

In this study, 4D flow CMR was coupled with a submaximal exercise challenge to examine the impact of preterm birth on right heart function in adolescents and young adults. Measurements in the MPA during exercise demonstrate lower SV and CI in preterm subjects relative to term controls. Likewise, measurements of KE efficiency and vorticity coupled with flow field visualizations in the RV during exercise suggested altered intraventricular flow in the preterm group. The early manifestations of these altered dynamics warrant further investigation given the young age of these subjects and their otherwise healthy appearance. Future studies are needed to further evaluate how these abnormal flow dynamics may progress to more severe disease in the preterm population.

## Supplementary Information

**Additional file 1.** Document describing methodology used to correct ECG gating files with skipped heart beats.

**Additional file 2.** Pathline animation of RV flow in a representative term subject.

**Additional file 3.** Pathline animation of RV flow in a representative preterm subject.

## Data Availability

The datasets used and analyzed during the current study are available from the corresponding author on reasonable request.

## Abbreviations

BMI: Body mass index
BSA: Body surface area
bSSFP: Balanced steady-state free precession
CI: Cardiac index
CMR: Cardiovascular magnetic resonance
ECG: Electrocardiogram
EDVI: End-diastolic volume index
EF: Ejection fraction
ESVI: End-systolic volume index
HR: Heart rate
KE: Kinetic energy
MPA: Main pulmonary artery
RV: Right ventricle/right ventricular
SNR: Signal-to-noise ratio
SV: Stroke volume
venc: Velocity encoding
VO_2max_: Maximal oxygen uptake
