# Supplementary material for: Exercise-induced irregular right heart flow dynamics in adolescents and young adults born preterm
Source: J Cardiovasc Magn Reson. 2021 Oct 21;23:116. doi: 10.1186/s12968-021-00816-2 (PMC8529801; doi:10.1186/s12968-021-00816-2)
Supplement: Supplementary file 1 — Additional file 1. Document describing methodology used to correct ECG gating files with skipped heart beats. [file 12968_2021_816_MOESM1_ESM.pdf]

Additional File 1 – ECG Gating Correction Algorithm

For the retrospective gating used in this manuscript, cardiac gating was established with four ECG leads connected to each subject's chest. The vector ECG is automatically analyzed by the physiological gating unit of the scanner, which records time stamps for each detected R-wave. These time fiducials are used to establish an average R-R interval (heartbeat length) and to assign a time position for each acquired MR echo within the cardiac cycle. Individual cardiac time frames can then be reconstructed by sharing data across heartbeats for identical cardiac intervals [1].

While ECG gating is normally very robust at rest, it was observed that cardiac triggers were missed for many subjects during exercise conditions. This can be seen in Figure A.1 below, which shows the recorded R-R interval length for each "heartbeat" over the course of an exercise scan.

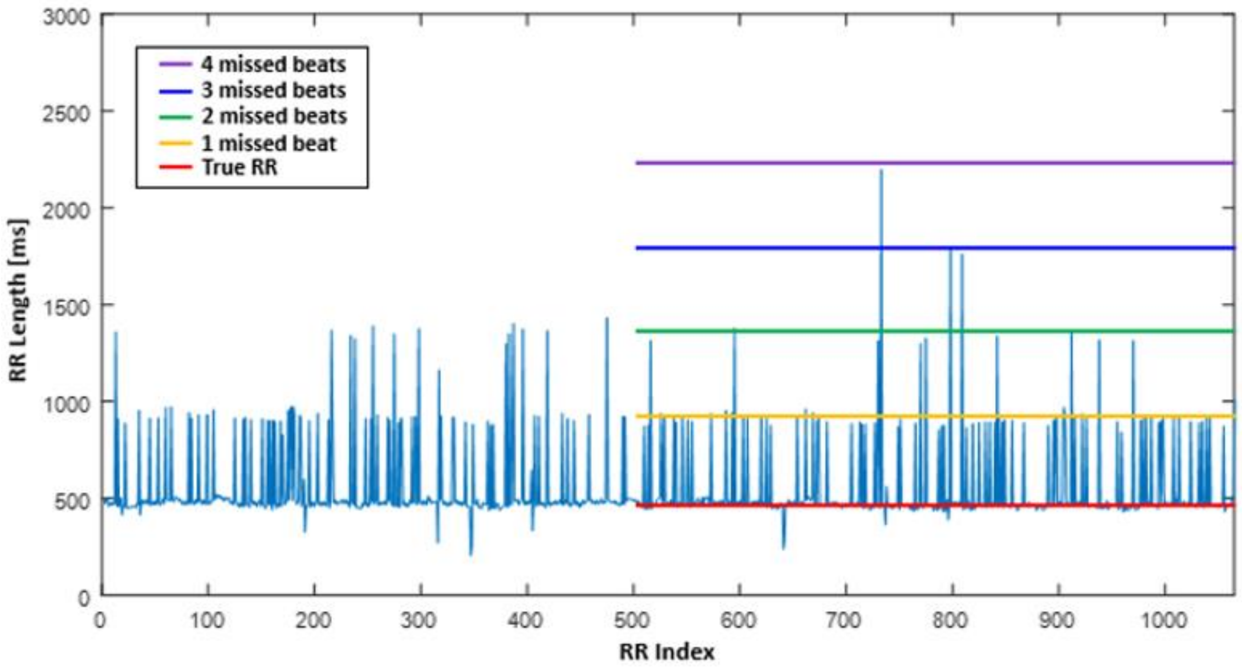

Figure A.1: All recorded R-R interval lengths by the ECG gating over the duration of a 10-minute exercise study. The true baseline interval length appears to be approximately 500 ms, but many interval lengths at near integer multiples of 500 ms can be observed where at least one previous R wave trigger was not detected by the gating.

As can be seen in Fig A.1, most R-R interval lengths were recorded to be approximately 500 ms long. However, one or multiple R wave triggers were missed in several instances, leading to recorded heartbeat lengths that were roughly integer multiple of 500 ms. In this example, the maximum recorded length corresponds to 4 consecutive missed triggers. It is likely that these missed triggers are a result of temporary losses in ECG signal quality due to subject movement and perspiration during exercise imaging.

With this type of processing, a large number of missed triggers can have a significant impact on the reconstructed flow waveforms as demonstrated in the blue flow curve in Figure A.2.

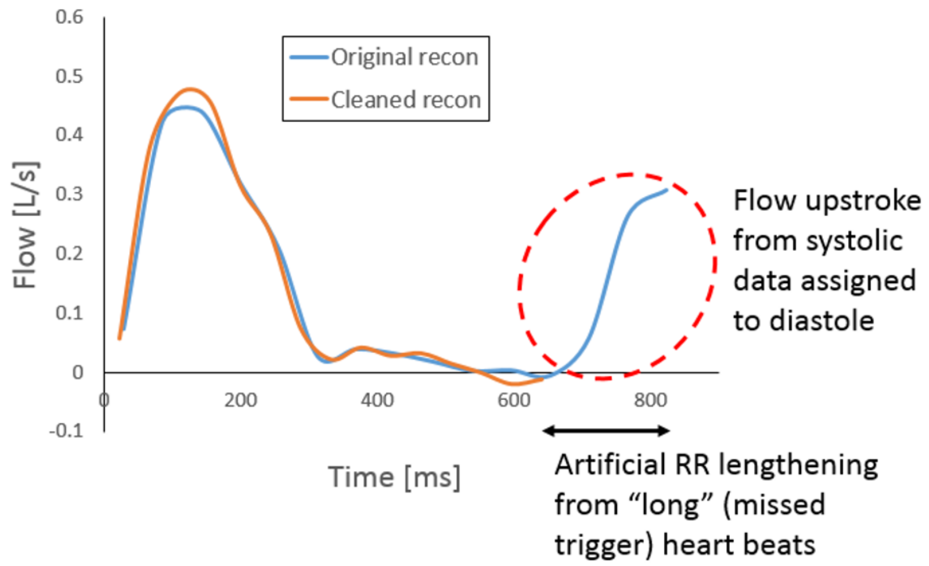

Figure A.2: (Blue curve) Original reconstructed aortic flow waveform with uncorrected ECG gating including many missed cardiac triggers. The inclusion of artificially long heartbeat lengths in the reconstruction increased the length of the reconstructed heartbeat. In addition, data corresponding to systole following a missed trigger was assigned to the end of the reconstructed heartbeat, resulting in a spurious, secondary systolic peak (dashed red circle). (Orange curve) Reconstructed aortic flow waveform with corrected gating. The heartbeat length has shortened, and the secondary systole flow has been eliminated, while preserving the original waveform characteristics.

There are 2 notable effects of multiple missed R-waves. The median of the recorded R-R intervals is longer than the physiological RR interval. In addition, data corresponding to systolic flow following a missed trigger is erroneously assigned to the end of the reconstructed heartbeat, creating a non-physiologic flow upstroke.

Here we applied a previously presented correction algorithm for the ECG timing information [2]. This MATLAB-based algorithm calculates each R-R interval length based on the recorded time stamps. Subsequently, a sliding window temporal filter compares each measured R-R interval length to the median value of the nearest 50 intervals. If the examined R-R interval length was approximately an integer multiple of the median filter value, the interval was subdivided into a new number of equal-length heart beats. While this approach assumes consecutive heartbeats are the same length, this was deemed acceptable due to the temporal “smearing” already inherent to using discrete cardiac phases when reconstructing data. When this algorithm was applied to Figure A.1, the resulting distribution shown in Figure A.3 was obtained. This algorithm successfully eliminated all missed triggers initially present in Figure A.1.

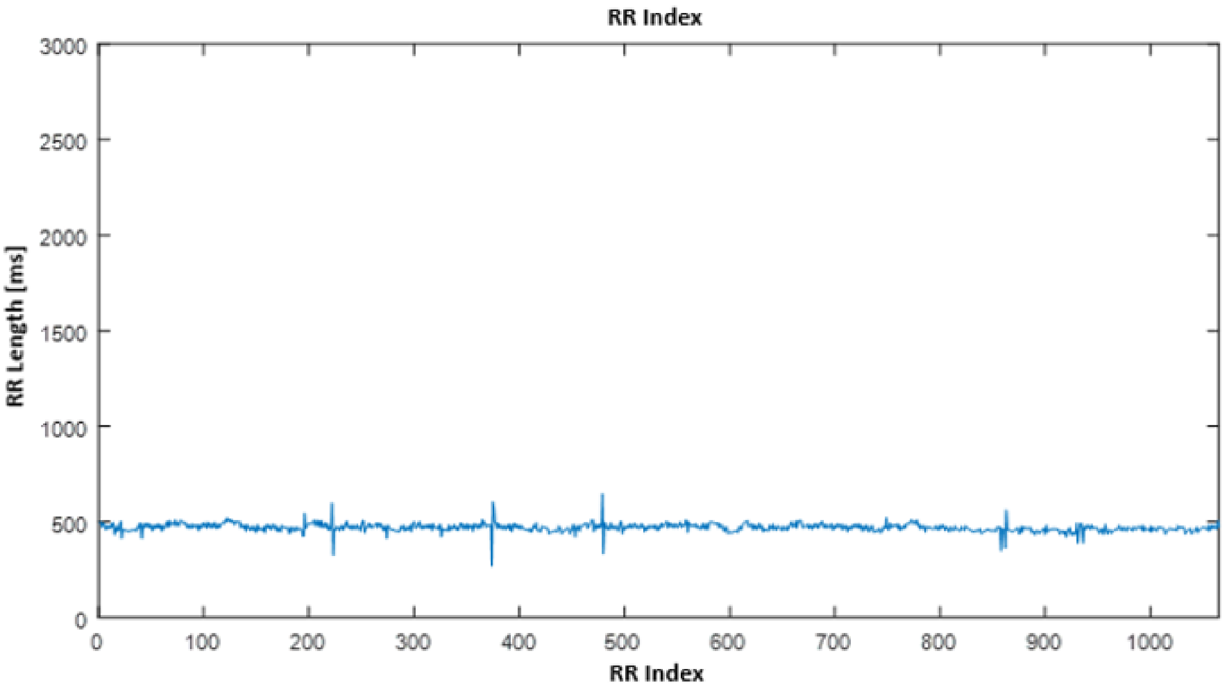

Figure A.3: RR-interval lengths after applying the ECG gating correction algorithm to the data in Figure A.1. All missed triggers have been successfully accounted for.

The effect of the algorithm on the reconstructed flow waveforms is shown by the orange curve in Figure A.2. The spurious secondary systole has been eliminated, and the reconstructed median heartbeat length has been properly shortened.

The MATLAB script for this algorithm is available from the corresponding author.

References

1. Lenz GW, Haacke EM, White RD. Retrospective Cardiac Gating: A Review of Technical Aspects and Future Directions. Magn Reson Imaging. 1989;7:445–55.
2. Macdonald J, Roberts G, Wieben O. ECG Characterization and Correction during Exercise Stress Imaging. Proc 26th Annu ISMRM Meet. 2018;
